# Acupuncture Enhances Dorsal Raphe Functional Connectivity in Knee Osteoarthritis With Chronic Pain

**DOI:** 10.3389/fneur.2021.813723

**Published:** 2022-01-18

**Authors:** Nan Gao, Haiping Shi, Sheng Hu, Bixiang Zha, Aihong Yuan, Jianhua Shu, Yinqiu Fan, Jin Bai, Hongyu Xie, Jingcheng Cui, Xiaoxiao Wang, Chuanfu Li, Bensheng Qiu, Jun Yang

**Affiliations:** ^1^Center for Biomedical Imaging, University of Science and Technology of China, Hefei, China; ^2^First Affiliated Hospital of Anhui University of Traditional Chinese Medicine, Hefei, China; ^3^School of Medical Information Engineering, Anhui University of Chinese Medicine, Hefei, China; ^4^Third People's Hospital of Hefei, Hefei, China

**Keywords:** functional magnetic resonance imaging, chronic pain, acupuncture, functional connectivity, knee osteoarthritis

## Abstract

**Introduction:**

Knee osteoarthritis is a common disease in the elderly. Patients suffer from long-term chronic pain and reduced life quality. Acupuncture has been proven to be an effective treatment for KOA. However, the neural mechanism of acupuncture is unclear, so far. Periaqueductal gray (PAG) and raphe nuclei (RPN) are essential structures associated with chronic pain in human brains. This study aims to investigate functional connectivity (FC) changes of PAG and RPN in KOA to interpret the neural mechanism of acupuncture.

**Methods:**

In 15 patients with KOA and 15 healthy controls (HC), we acquired Visual Analog Scale (VAS) scores and resting-state fMRI images of each participant before and after acupuncture stimulation on EX-LE5 acupoint. Then, PAG and RPN were selected as seeds to perform FC analysis based on resting-state fMRI images. Finally, we compared FC patterns of PAG and RPN between patients with KOA and HC, then between pre-acupuncture and post-acupuncture. Correlations between FC values and VAS scores were calculated as well.

**Results:**

For PAG, FC of patients with KOA was lower in the right lingual gyrus at post-acupuncture compared with HC (*p* <0.001, uncorrected). For dorsal RPN, FC of patients with KOA was significantly higher in right putamen at post-acupuncture compared with HC (*p* <0.001, corrected with FDR), and FC changes were significant between pre-acupuncture and post-acupuncture in patients with KOA. Post-acupuncture FC values between dorsal RPN and right putamen were correlated with VAS scores. For medial RPN, FC of patients with KOA was lower in the right cerebellum at post-acupuncture compared with HC (*p* <0.001, uncorrected), but no significant FC changes were found between pre-acupuncture and post-acupuncture in patients with KOA. FC values between medial RPN and right cerebellum were not correlated with VAS scores at pre-acupuncture and post-acupuncture.

**Discussion:**

Our study demonstrated that acupuncture enhanced FC between dorsal RPN and the right putamen in patients with KOA, which was associated with chronic pain intensity. This result suggests that acupuncture stimulation can enhance FC between dorsal raphe and striatum, illustrating a neural mechanism that acupuncture can drive the patients' brain, with KOA, to perceive pain.

## Introduction

Knee osteoarthritis is a common type of arthritis and prevalently affects the quality of life among the elderly (1). Chronic pain is the primary complaint associated with KOA, severely influencing the patients by physical dysfunction and muscular weakness (2, 3). Acupuncture is considered an effective therapy to relieve the chronic pain of KOA (4–6), but the mechanism underlying its clinical efficacy is still controversial. Previous studies have reported that chronic pain can induce changes in brain function (7–9), and acupuncture stimulation can mediate brain function to relieve chronic pain (10). Therefore, this study aims to explore the mechanism of acupuncture treatment by investigating how acupuncture induces the functional connectivity (FC) of brains in patients with KOA.

Periaqueductal gray and raphe nuclei, located in the brainstem, play a well-established pain responses because these structures have optimal anatomical positioning to integrate relevant contextual information, driving the central neural system to release pain-related neurotransmitters (11, 12). Recently, functional MRI (fMRI) studies have investigated how the activation of PAG and RPN contributes to neural mechanisms underlying chronic pain. Lee et al. applied the FC method to test the FC changes between patients with chronic migraine and with episodic migraine. They revealed that FC was enhanced, from PAG and RPN to cortical regions, in patients with chronic migraine (13). Notably, a recent study on the mouse model of OA has found that, in the early phase of OA pain, the PAG network, especially the connection between PAG and RPN, may contribute to the transition from acute to chronic OA pain (14). Another study demonstrated that dorsal RPN and ventral PAG have the potential to regulate pain by releasing dopamine and glutamate (15).

Acupuncture, one of the traditional Chinese treatment modalities, has a good therapeutic effect on chronic pain (16), that includes chronic migraine and chronic low back pain. Some previous studies reported that the possible mechanism of relieving pain by acupuncture stimulation was mediating through central pain modulation of the PAG and RPN in the brainstem (17, 18). Although some studies have supported that acupuncture is an efficient treatment tool for KOA pain, the neural mechanism underlying acupuncture treatment on KOA is unclear. Therefore, this study aimed to clarify the acupuncture neural mechanism on KOA by using fMRI. Considering the above, we speculated that acupuncture stimulation could alter FC of PAG and RPN in patients with chronic KOA pain, which is associated with pain-related clinical symptoms.

To test this hypothesis, we respectively calculated FC for patients with KOA and healthy controls (HC) at pre-acupuncture and post-acupuncture stimulation. The group differences between KOA and HC, and between pre-acupuncture and post-acupuncture, were evaluated, as well as the correlations between altered FC and the related clinical symptom were further calculated to assess how the FC changes through acupuncture stimulation have modulated chronic KOA pain.

## Materials and Methods

### Participants

Fifteen patients with chronic KOA pain (mean age: 59.13 ± 10.27; eight females), whose pain lasts at least 4 months, were recruited from the First Affiliated Hospital of Anhui University of Chinese Medicine (AUCM), while 15 HC (mean age: 58.53 ± 8.15; 11 females) were recruited from the local community. The patients who presented with neurological or psychiatric diseases, poorly controlled hypertension, head injury, or any other conditions that might affect the study were excluded. All the participants had signed an informed consent before the study, which was approved by the Ethics Committee of the First Affiliated Hospital of AUCM (No. 2021AH-29).

### KOA Diagnostic Criteria

The diagnostic criteria of KOA were determined according to the Chinese Guideline for Diagnosis and Treatment of Osteoarthritis (2021 edition) formulated by the Joint Surgery Branch of the Chinese Orthopedic Association (19). Patients with KOA were diagnosed by the results of both clinical and X-ray examinations, plus the following rules: (1) middle-aged and elderly people; (2) recurrent knee pain for nearly a month; (3) morning stiffness ≤ 30 min; and (4) bone frictions when physically exercising.

### Pain Assessment

Pain intensity was evaluated by the Visual Analog Scale (VAS), a tool widely used to measure pain (20). The patients were asked to indicate their perceived pain intensity as a point along a 100-mm horizontal line, and the length from the left edge to this point was measured to acquire a VAS score. The VAS score ranges from 0 to 10, where VAS = 0 represents the patient feels no pain, VAS < 3 indicates the patient feels mild but tolerable pain, 4 < VAS < 6 means the patient feels pain and sleep is affected, and 7 < VAS < 10 depicts the patient feels severe pain, which is unbearable and already affects the sleep and appetite. Detailed information is presented in Supplementary Table 1.

### Experiment Design

Pre-acupuncture resting-state fMRI data were acquired with 205 time points for 6 min 50 s before acupuncture stimulation. After that, acupuncture stimulation was performed at EX-LE5 acupoint, which is considered effective in treating KOA (21). When the participants received *de-qi* sensation (soreness, numbness, fullness, and heaviness) (22), task-state fMRI data with 205 time points were acquired for 6 min 50 s. Finally, after pulling out the acupuncture needles, post-acupuncture resting-state fMRI data acquisition of 205 time points was obtained for 6 min 50 s. The workflow is shown in Supplementary Figure 2. For the patients with KOA, VAS scores were acquired for assessment of pain intensity at both pre-acupuncture and post-acupuncture. In this study, we used the pre-acupuncture and post-acupuncture resting-state data and the VAS scores for analysis.

### Image Acquisition

MRI data were acquired using a 3.0-Tesla MRI scanner (Discovery MR750, General Electric, USA), with an eight-channel high-resolution radio-frequency head coil. Sagittal 3D T1-weighted images were acquired using T1-3D BRAVO sequence with repetition time (TR)/echo time (TE): 8.16 ms/3.18 ms, flip angle (FA): 12°, matrix: 256 × 256, field of view (FOV): 256 × 256 mm, slice thickness: 1 mm, with 170 axial slices with no gap. Resting-state and task-state fMRI data were acquired using a gradient-echo single-shot echo planar imaging sequence with TR/TE: 2,000/30 ms, FOV: 220 × 220 mm, matrix: 64 × 64, FA: 90?, slice thickness: 3 mm, with 205 volumes. All the participants were instructed to lie down with their eyes closed, keep their minds relaxed, and not fall asleep during the scanning.

### Data Preprocessing

For each participant, resting-state fMRI images were preprocessed using a combination of analysis packages, including FSL and AFNI. Functional images were preprocessed using the following steps: (1) the first 10 functional images were discarded to eliminate transients and account for T1 relaxation effects, followed by slice timing to compensate for acquisition delays across slices; (2) motion correction was performed by realigning all functional images to the middle image, and the data with head motion over 2 mm or 2° were excluded; (3) functional images were co-registered to the high-resolution anatomical images, and then normalized to Montreal Neurological Institute standard brain; (4) voxels were re-sampled to 2 × 2 × 2 mm^3^ resolution; (5) images were spatially smoothed with a 6-mm full width at half-maximum (FWHM) Gaussian kernel; (6) the data were linearly detrended, and the residual signals were band-pass temporal filtered at.01–0.1 Hz; and (7) nuisance variable regression was performed to regress out the six head motion parameters, as well as the signals of white matter (WM) and cerebrospinal fluid (CSF).

### Functional Connectivity Analysis

**Figure 1:**
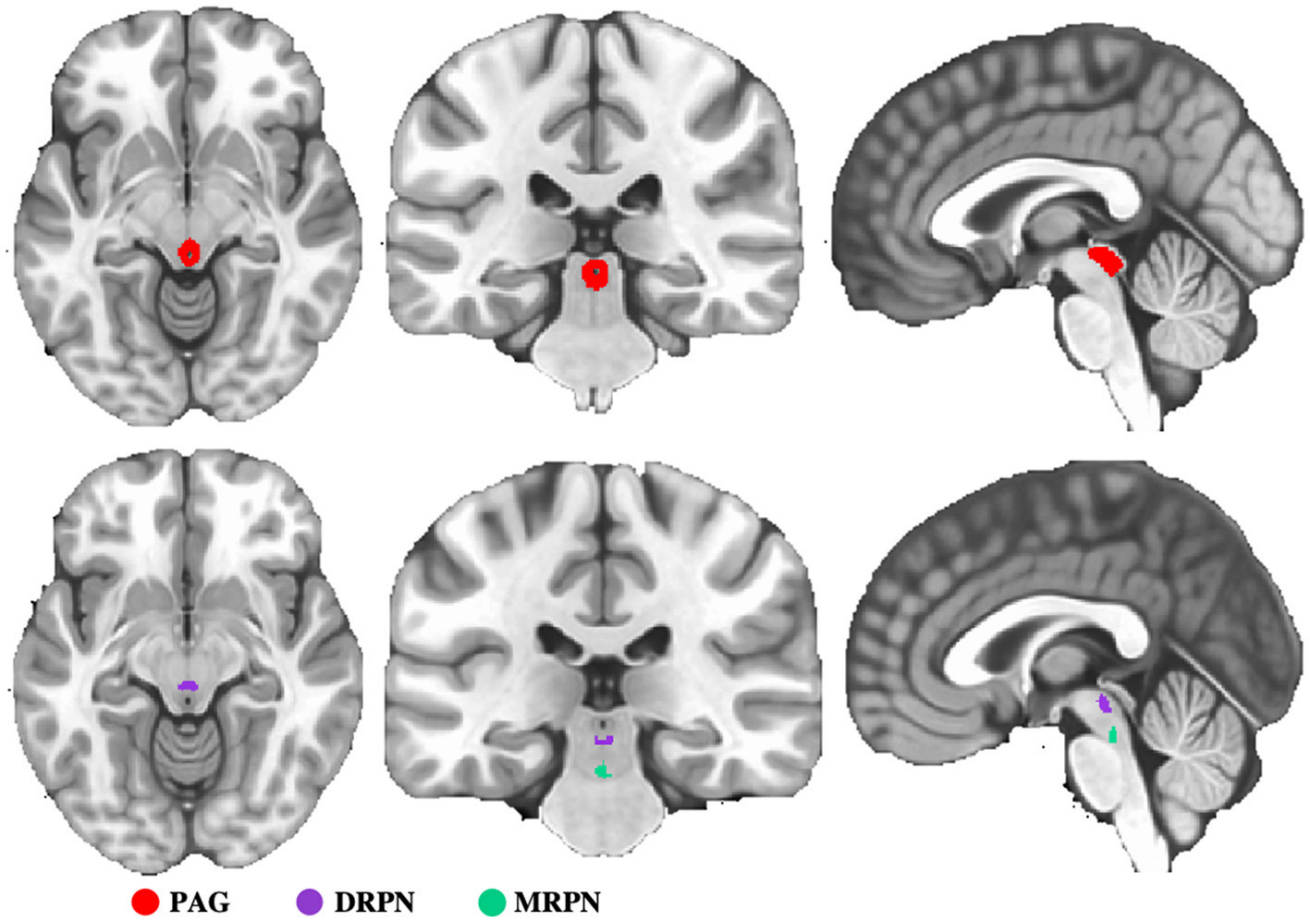
Representation of seed regions. PAG, periaqueductal gray; DRPN, dorsal raphe nuclei; MRPN, medial raphe nuclei.

To evaluate FC changes of PAG and RPN, we first selected these two regions as seeds for FC analysis. Seed of PAG was structurally defined with the “atlas of the basal ganglia” (https://www.nitrc.org/projects/atag/), in which the nonlinear elderly atlas of PAG was selected for FC analysis. RPN was divided into dorsal and medial sub-regions, defined based on a previous study (23). The seeds of PAG and RPN are shown in Figure 1.

For each participant, the Pearson correlation coefficient between the mean time series of each seed and the time series of each voxel across the whole brain was calculated. Then, the coefficients were converted to a z-value using Fisher r-to-z transformation to improve the normality.

### Statistical Analysis

A paired two-sampled *t*-test was applied to both KOA and HC groups to compare FC changes between pre-acupuncture and post-acupuncture, uncovering how acupuncture mediates FC. An unpaired two-sampled *t*-test was used at both pre-acupuncture and post-acupuncture to compare FC changes between KOA and HC to investigate the group differences mediated by acupuncture. The results of group analysis were corrected using a false discovery rate (FDR) of *p* < 0.001.

### Correlation Analysis

We explored the relationships between FC values and the VAS scores in the patient group to identify how acupuncture affects chronic pain through mediating brain function. For all correlation analyses, we used partial correlations to factor out age and sex.

## Results

### FC Changes of PAG

No FC changes of PAG were observed between pre-acupuncture and post-acupuncture in both patients with KOA and groups with HC. No FC differences of PAG were found between the two groups at pre-acupuncture. Lower FC of PAG in patients with KOA was found in right lingual gyrus at post-acupuncture compared with HC (Supplementary Figure 1 and Table 1, *p* < 0.001, uncorrected).

**Table 1:** Group differences of FC between KOA and HC at post-acupuncture.

**Regions**	**MNI**			**Z Value**	**Voxel size**
	**x**	**y**	**z**		
**Periaqueductal gray**					
Right Lingual Gyrus	4	−64	−2	−4.16	42
**Dorsal Raphe**					
Right Putamen	26	0	6	4.97	91
**Medial Raphe**					
Right Cerebellum	16	−72	−35	−4.65	41

*FC between dorsal raphe and right putamen was corrected for FDR with p < 0.001, whereas FC between periaqueductal gray and right lingual gyrus, and also between medial raphe and right cerebellum were uncorrected with p < 0.001*.

### FC Changes of RPN

**Figure 2:**
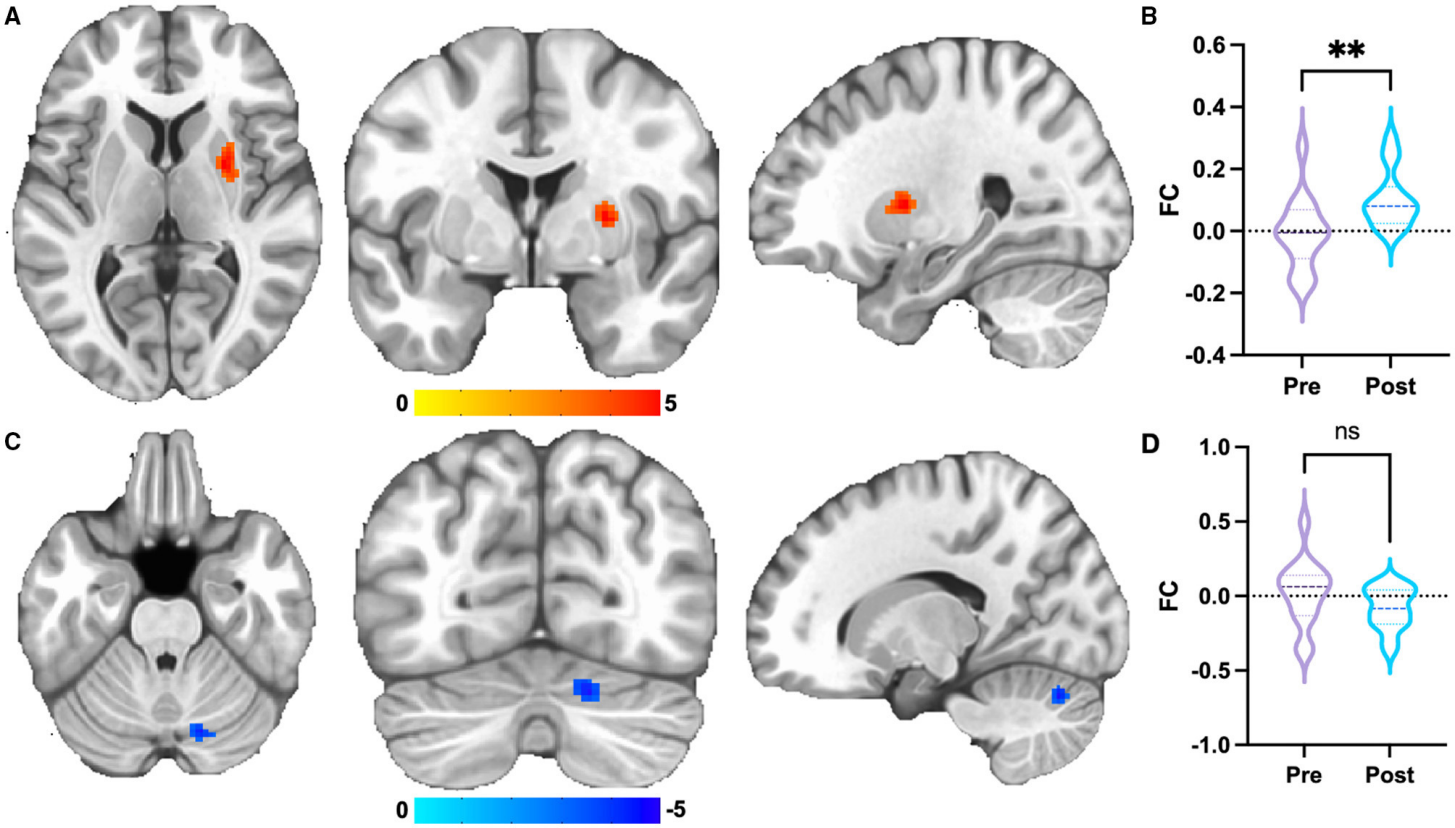
Group differences of FC. **(A)** FC of dorsal RPN of patients with KOA enhanced at post-acupuncture compared with HC. The result was corrected for FDR with *p* <0.001. **(B)** FC differences of right putamen between pre-acupuncture and post-acupuncture in KOA. **(C)** FC of medial RPN of patients with KOA decreased at post-acupuncture compared with HC. The result was uncorrected with *p* < 0.001. **(D)** FC differences of right cerebellum between pre-acupuncture and post-acupuncture in KOA. **: represents significant group difference with *p* <0.05; ns: represents no significant group difference.

**Figure 3:**
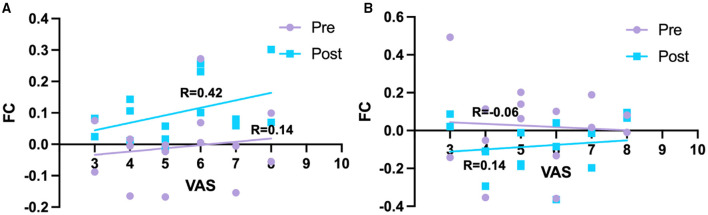
Relationships between FC and pain intensity. **(A)** Correlation between FC of right putamen and pain intensity in KOA. **(B)** Correlation between FC of right cerebellum and pain intensity in KOA. Pre, pre-acupuncture; Post, post-acupuncture.

For dorsal RPN, no FC changes were observed between pre-acupuncture and post-acupuncture in two groups; no FC differences were found between patients with KOA and HC at pre-acupuncture, whereas FC in patients with KOA was significantly higher in right putamen at post-acupuncture compared with HC (Figure 2A and Table 1, *p* < 0.001, corrected with FDR). The FC difference of right putamen was significant between pre-acupuncture and post-acupuncture in patients with KOA (Figure 2B). Post-acupuncture FC values of right putamen were correlated with VAS scores, while the pre-acupuncture FC values were not (Figure 3A).

For medial RPN, no FC changes were observed between pre-acupuncture and post-acupuncture of two groups; no FC differences were found between patients with KOA and HC at pre-acupuncture, whereas FC in patients with KOA was lower in right cerebellum at post-acupuncture compared with HC (Figure 2C and Table 1, *p* < 0.001, uncorrected). FC difference of right cerebellum was not significant between pre-acupuncture and post-acupuncture in patients with KOA (Figure 2D). FC values of right cerebellum were not correlated with VAS scores at both pre-acupuncture and post-acupuncture (Figure 3B).

## Discussion

The brainstem involvement in relieving chronic pain has been investigated in previous studies, demonstrating a mediating role of PAG and RPN, as well as their interactions with cortical regions (24, 25). Therefore, we applied the resting-state fMRI to evaluate the neural mechanism underlying acupuncture, intervening in chronic KOA pain by investigating the FC changes of the PAG and RPN mediated by acupuncture. Our results suggested that acupuncture enhanced FC between dorsal RPN and the right putamen in patients with KOA, and enhanced FC is associated with chronic pain intensity.

Periaqueductal gray (PAG) plays a central role in the descending modulation of pain (26). Dysfunction of PAG is thought to contribute to dysregulation of pain, which is the main reason for chronic pain (24). Previous studies have demonstrated that FC between PAG and prefrontal cortex/anterior cingulate cortex in low back chronic pain (27) and chronic migraine (28) is enhanced after acupuncture treatment, associated with the relieving of pain symptoms (28). However, in our study, FC differences of PAG were not found between patients with KOA and HC. Acupuncture stimulation has also not induced PAG-related FC changes in patients with KOA with chronic pain. These results were inconsistent with previous studies. In this study, the patients also received several other medications in addition to acupuncture treatment, which possibly drove the FC of PAG close to the normal level. Previous studies have demonstrated that transient drug therapy could enhance the patients' brain function, whereas the patients were also accompanied by clinical symptoms (29, 30). This is the possible explanation why we did not observe the changes in PAG function in the patients with chronic KOA pain.

Previous studies have interpreted the neural mechanism of dorsal RPN in the regulation and the perception of pain from electrophysiology and neuroimaging, based on the fact that FC between dorsal RPN and cortical/subcortical regions was increased in patients with chronic pain after treatment (13, 16). The striatum receives serotonin innervation from the dorsal RPN, which implicates the behaviors of emotions, depression, and anxiety (31, 32). This evidence was further proved in a PET-fMRI study, revealing that dorsal RPN has strong functional connections with striatum (23). In present studies, FC between dorsal RPN and right putamen was enhanced after acupuncture stimulation rather than FC between medial RPN and right cerebellum. Moreover, the rising value of FC was associated with pain intensity. In summary, our results may explain that acupuncture stimulation induces the enhanced FC between dorsal RPN and striatum to drive the patients' brain with KOA to perceive the pain.

There were also several limitations in this study. The sample size of the participants was relatively small so that individual variation may affect the accuracy of the results. Moreover, considering the comfort and cooperation of the patients, short-term acupuncture stimulation was conducted in this research rather than typical acupuncture therapy, because the participants were lying in the bore of MRI equipment through the whole experiment. Our study pursued the instantaneous effect of how the brain perceives the pain right after the acupuncture stimulation; not the neural mechanism of the acupunctural therapeutic effect. For further research, more participants will be recruited, and we will simultaneously study the effects of both the short-term acupuncture stimulation and long-term acupuncture therapy.

## Conclusions

In conclusion, our results have demonstrated that acupuncture stimulation can enhance FC between dorsal raphe and striatum, illustrating a neural mechanism that acupuncture can drive the patients' brain, with KOA, to perceive pain.

## Data Availability Statement

The raw data supporting the conclusions of this article will be made available by the authors, without undue reservation.

## Ethics Statement

The studies involving human participants were reviewed and approved by the Ethics Committee of the First Affiliated Hospital of Anhui University of Chinese Medicine. The patients/participants provided their written informed consent to participate in this study.

## Author Contributions

NG and HS wrote the first draft of the article, edited, and revised the article. BZ, AY, JS, and YF analyzed imaging data. JB, HX, and JC contributed to data acquisition. SH, XW, CL, BQ, and JY designed the study. All the authors contributed to and have approved the final version of the article.

## Funding

This work was supported by the National Natural Science Foundation of China (No. 81873370), Ministry of Science and Technology of the People's Republic of China (No. 2018YFC170460-4), the Support Project of Natural Science Foundation for Distinguished Young Scholars of Anhui Province (No. gxyq2020016), and the Key Project of Natural Science Foundation of Anhui Province (No. KJ2020A0394).

## Conflict of Interest

The authors declare that the research was conducted in the absence of any commercial or financial relationships that could be construed as a potential conflict of interest.

## Publisher's Note

All claims expressed in this article are solely those of the authors and do not necessarily represent those of their affiliated organizations, or those of the publisher, the editors and the reviewers. Any product that may be evaluated in this article, or claim that may be made by its manufacturer, is not guaranteed or endorsed by the publisher.
