# Supplementary material for: Acupuncture Enhances Dorsal Raphe Functional Connectivity in Knee Osteoarthritis With Chronic Pain
Source: Front Neurol. 2022 Jan 18;12:813723. doi: 10.3389/fneur.2021.813723 (PMC8805588; doi:10.3389/fneur.2021.813723)
Supplement: Supplementary file 1 [file Data_Sheet_1.pdf]

Knee OA	Age	Gender	VAS	HC	Age	Gender
Subject 1	49	M	5	Subject 1	49	M
Subject 2	56	F	3	Subject 2	54	F
Subject 3	71	M	6	Subject 3	57	F
Subject 4	41	M	5	Subject 4	49	F
Subject 5	58	F	4	Subject 5	69	F
Subject 6	52	F	6	Subject 6	67	F
Subject 7	70	F	8	Subject 7	58	F
Subject 8	58	F	7	Subject 8	51	F
Subject 9	58	F	6	Subject 9	56	M
Subject 10	69	F	7	Subject 10	75	M
Subject 11	70	M	8	Subject 11	49	F
Subject 12	58	M	4	Subject 12	69	F
Subject 13	71	M	3	Subject 13	59	F
Subject 14	65	F	5	Subject 14	60	M
Subject 15	41	M	4	Subject 15	56	F

Supplementary Table 1. Clinical information of all subjects. Knee OA: Knee osteoarthritis; HC: healthy control; VAS: Visual Analogue Scale; M: male; F: female.

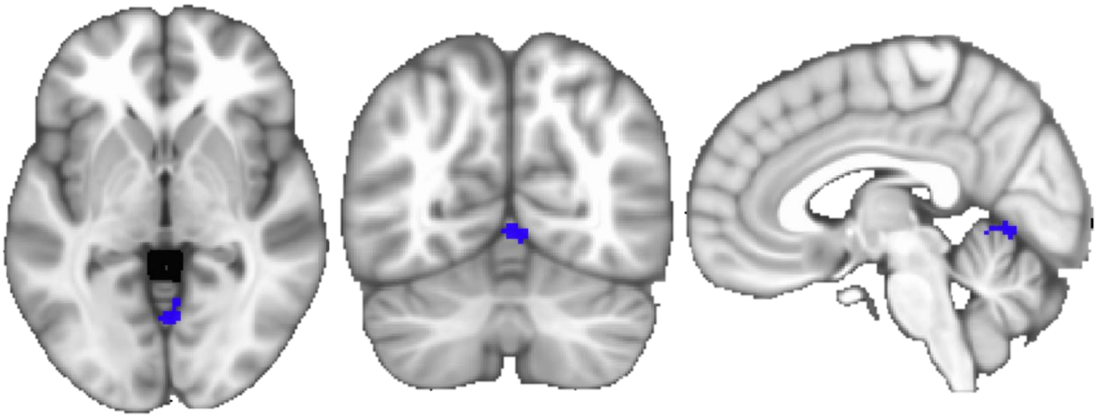

Supplementary Figure 1. Group differences of FC of PAG. KOA patients' FC of PAG was lower in right lingual gyrus at post-acupuncture compared with HC.

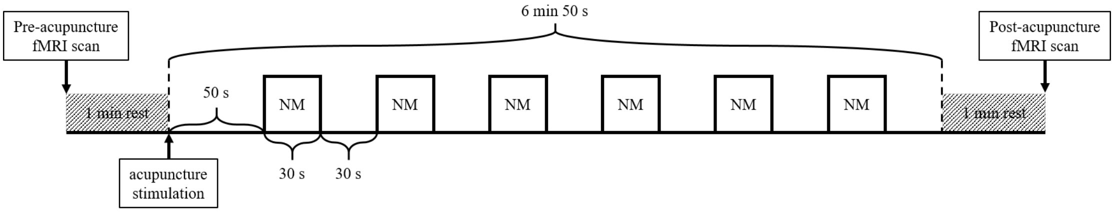

Supplementary Figure 2. Workflow of experiment design in this study.
